# Misrepresentation of Overall and By-Gender Mortality Causes in Film Using Online, Crowd-Sourced Data: Quantitative Analysis

**DOI:** 10.2196/70853

**Published:** 2025-06-24

**Authors:** Calla Glavin Beauregard, Christopher M Danforth, Peter Sheridan Dodds

**Affiliations:** 1 Computational Story Lab, University of Vermont, Burlington, VT, United States; 2 MassMutual Center of Excellence, Vermont Complex Systems Institute, University of Vermont, 89 University Place, Burlington, VT, 05452, United States, 1 (802) 656-3392; 3 Department of Mathematics and Statistics, University of Vermont, Burlington, VT, United States; 4 Vermont Advanced Computing Center, University of Vermont, Burlington, VT, United States; 5 Department of Computer Science, University of Vermont, Burlington, VT, United States; 6 Santa Fe Institute, Santa Fe, NM, United States

**Keywords:** media representation, gender, mortality, natural language processing, NLP, data science

## Abstract

**Background:**

The common phrase “representation matters” asserts that media has a measurable and important impact on civic society’s perception of self and others. The representation of health in media, in particular, may reflect and perpetuate a society’s disease burden.

**Objective:**

In this study, for the top 10 major causes of death in the United States, we aimed to examine how cinematic representation overall and by-gender mortality diverges from reality.

**Methods:**

Using crowd-sourced data on over 68,000 film deaths from Cinemorgue Wiki, we employ natural language processing techniques to analyze shifts in representation of deaths in movies versus the 2021 National Vital Statistics Survey top 10 mortality causes. We parsed, stemmed, and classified each film death database entry, and then categorized film deaths by gender using a specifically trained gender text classifier.

**Results:**

Overall, movies strongly overrepresent suicide and, to a lesser degree, accidents. In terms of gender, movies overrepresent men and underrepresent women for nearly every major mortality cause, including heart disease and cerebrovascular disease (chi-square test, *P*<.001); 73.6% (477/648) of film deaths from heart disease were men (vs 384,866/695,547, 55.4% in real life) and 69.4% (50/72) of film deaths from cerebrovascular disease were men (vs 70,852/162,890, 43.5% in real life). The 2 exceptions for which women were overrepresented are suicide and accidents (chi-square test, *P*<.001), with 39.7% (945/2382) deaths from suicide in film being women (vs 9825/48,183, 20.4% in real life) and 38.8% (485/1250) deaths from accidents in film being women (vs 75,333/225,935, 33.5% in real life).

**Conclusions:**

We discuss the implications of under- and overrepresenting causes of death overall and by gender, as well as areas of future research.

## Introduction

Cardiovascular disease is the number one cause of death in both men and women in the United States according to the National Survey of Vital Statistics (NVSS), published annually by the Centers for Disease Control and Prevention (CDC) [1]. However, men and women can experience different risk factors related to cardiovascular disease, as well as present different symptoms when experiencing a cardiovascular event [2]. In recent years, the American Heart Association has highlighted the importance of understanding these differences with calls for an overhaul in how physicians consider gender in the diagnosis of cardiovascular disease [3].

Representation in media is central to how we perceive our society, including health. Media portrayal and representation influence thoughts, behaviors, and actions toward certain groups, ideas, and phenomena, that is, the “[m]edia, in short, [is] central to what ultimately come to represent our social realities” [4]. This connection has been studied across many demographic categories and aspects of social reality. Broadly, narratives delivered by film and other forms of media influence beliefs, which has been theorized as a “dual empathy” pathway wherein viewers process their feelings and thoughts while also perspective sharing with fictional characters [5]. Salient media stories in both film and books impact identity formation in both middle-age and young adults when relating their own life stories [6].

A subset of narrative theory is transportation theory, which purports that individuals who experience greater narrative transportation in response to fictional and nonfictional media are more likely to change their behaviors and beliefs and identify fewer factual inconsistencies in that media [7]. Research has explored the connection of transportation in film with a variety of health conditions and behaviors; the depiction of smoking in entertainment film increased tobacco use behaviors in adolescents [8], public awareness films improved young adult attitudes toward older adults [9], drunk driving film scenarios reduced self-risk assessment in drunk-driving [10], and a film depicting individuals with taboo chronic gut conditions improved understanding of the conditions and increased likeliness of discussing these conditions with friends and family pre- and postsurvey [11]. In summary, film depictions influence viewer perception and beliefs across areas of health.

Outside of transportation theory, there is ample research regarding the media’s representation and impact on health via other pathways. A recent study examined how acute myocardial infarction (eg, a heart attack) was portrayed in 100 popular films, demonstrating the general inaccuracy of its depiction of symptoms as well as its depiction by gender [12]. Aside from that study, in terms of gender demographics, most research tends to focus on negative representation of woman in media, specifically in terms of sexual objectification; “very few studies appear to be available on the relationship between media representation and non-sexual objectification” of women [13]. Similarly, many aspects of social reality, such as economic, health, and well-being outcomes, have been studied with respect to media representation (although not explicitly through the lens of gender). For example, there is a rich history of research on how representation in the media influences the commission of intentional self-harm [14]. In summary, the media typically does not accurately portray official suicide data, and the most comprehensive systematic review on the topic links media reporting and suicidality, with current evidence suggesting increased suicidality trends with increased reporting of suicide [14].

Aside from suicidality, there is a diversity of studies on health issues and their media depiction; a nonexhaustive survey of the literature includes a study on the depiction of obesity in media and mortality in Indigenous Australians [15], a study of how bisexual individuals’ mental health is impacted by depictions of bisexual people in media [16], and globally, a scoping review on how media representation of major corporations influence noncommunicable disease risk assessment and public health policy-making related to consumer products [17]. Thus, while there is research on how narrative influences health [5-8], and research on health representation and gender representation in media [18-23], there is little research that explores the overlap between health outcomes and gender representation in media, a gap we aimed to address in this study.

To examine the connection between media and mortality in this study, we downloaded counts by year of all-time cinematic deaths (referred to here as film deaths) from the crowd-sourced Cinemorgue Wiki database [24] and compared them with the 2021 NVSS [1] counts of top 10 causes of mortality by gender. Using natural language processing, we extracted the Cinemorgue counts, classified the deaths by gender, and consolidated these data against the NVSS counts. We then analyzed the comparative ratios by gender between real life and film and considered significant differences in these ratios using the chi-square test. The aim of this study is to understand the extent to which cinematic deaths in Cinemorgue reflect real-world mortality by gender and cause of death.

## Methods

There are 2 main data sources for this project: the Cinemorgue Wiki database of film deaths [24] and the 2021 NVSS Leading Causes of Mortality Report [1].

### Collecting and Cleaning Data

First, we identified all-time film deaths using the Cinemorgue Wiki [24] which is a crowd-sourced database that allows any members of Fandom to update death by cause by film and film year (1895‐2023), with a short description of the death. The site is organized by death type or by actor (not by character) and each entry is a short 1‐2 sentence description of the film death. It is inclusive of any movies publicly entered, and thus not limited to American film deaths only. Conveniently, Cinemorgue has historically provided an .xml download of their entire website (as of August 16, 2023) and other researchers have previously classified cinematic deaths using Federal Bureau of Investigation (FBI) homicide specifications [25]. Refer to Table 1 for a basic description of the database entries.

Using this existing script, we adjusted the classifying portion to identify deaths according to the CDC NVSS’s top mortality causes by year for 2021 [1]. Our script parsed the description of each film and reduced it to word stems by film entry. We used generally broad word stems that captured the colloquial terms describing different mortality causes (eg, “heart attack” and “heart failure”). We then categorized film deaths by gender using a reasonably comprehensive and inclusive classifier from PyPi project gender-guesser, which was trained on a representative international database of names that were independently validated by native speakers of many languages [26]. When applying gender-guesser in this study, all 40,000 plus names were included to account for various languages and ethnic backgrounds of actors represented in film (the Cinemorgue Wiki includes movies from many different countries). Furthermore, the code allowed for the classification of mostly male, mostly female, androgynous, and unknown names. Table 2 enumerates the initial name classification.

Based on a random manual review of classification, “mostly male” and “mostly female” seemed to accurately categorize well-known actors, “mostly male” was grouped with male, and “mostly female” was grouped with female. Based on the size of the data and this classifier, we removed androgynous or unknown names (collectively referred to here as “other”), discussed in the Limitations section. The 2021 top 10 leading causes of death in the United States are depicted in Table 3 [1]. This list actually comprises 13 leading causes of death because the ninth and tenth leading causes of death by gender differ, and a category of “other” is including to reflect the remaining causes. If absent from the official report, these data were queried directly from the CDC WONDER database [27] using the same *International Classification of Diseases, Tenth Revision* (*ICD-10*) parameters from the NVSS [1]. We calculated the ratio of mortality by gender by performing a simple ratio of male or female deaths by mortality cause over total deaths by that cause. It is important to note that the top 10 leading mortality causes differ year-to-year, and 2021 was the second year to include COVID-19 deaths. In addition, the medical terms describing these causes of death are extensive and often highly technical; for ease of understanding, we provide additional notations to specify the colloquial terms for these diseases.

**Table 1.** All film death entries by year percentiles as per Cinemorgue (total deaths=68,535).

Percentile	Year
Minimum	1895
25th	1976
50th	1995
75th	2010
Maximum	2023

**Table 2.** Results of the gender classifier, gender-guesser, used on all Cinemorgue data, before and after consolidation.

Gender	Count before, n	Count after, n
Women	15,673	17,451
Men	39,627	42,458
Unknown	7383	—^a^
Mostly men	2831	—
Mostly women	1778	—
Androgynous	1243	—
Nonmale or nonfemale^b^	—	8626

a Categories collapsed after consolidation.

b For the purposes of this table, the terms “male” and “female” are used to be consistent with the name classification package configuration.

**Table 3.** Percentage by gender of the National Vital Statistics System 2021 leading causes of mortality in the United States.

Mortality cause, type (women rank, men rank)	Total, n	Female, n (%)		Male, n (%)	
Diseases of the heart (1, 1)	695,547	310,661 (44.6)		384,886 (55.4)	
Malignant neoplasm, cancer^a^ (2, 2)	605,213	286,543 (47.3)		318,670 (52.7)	
COVID-19 (3, 3)	416,893	180,283 (43.2)		236,610 (56.8)	
Accidents, unintentional injuries (6, 4)	224,935	75,333 (33.5)		149,602 (66.5)	
Cerebrovascular disease, stroke^a^, brain hemorrhage^a^ (4, 5)	162,890	92,038 (56.5)		70,852 (43.5)	
Chronic lower respiratory disease, COPD^a^^b^, emphysema^a^ (7, 6)	142,342	74,814 (52.6)		67,528 (47.4)	
Alzheimer disease (5, 8)	119,399	82,424 (69)		36,975 (31)	
Diabetes mellitus (8, 7)	103,294	44,666 (43.2)		56,628 (54.8)	
Chronic liver disease and cirrhosis (11, 9)	56,585	20,878 (36.9)		35,707 (63.1)	
Nephritis (9, 10)	54,358	25,769 (47.4)		28,589 (52.6)	
Suicide, intentional self-harm (15, 8)	48,183	9825 (20.4)		38,358 (79.6)	
Essential hypertension (10, 15)	42,816	22,730 (53.1)		20,086 (46.9)	
Other	791,776	400,159 (50.5)		391,617 (49.5)	

a Colloquial term.

b COPD: chronic obstructive pulmonary disease.

### Analysis of Gender Ratios: Visualization and Chi-Square Testing

In both datasets, we calculated the ratio of mortality by gender by cause by performing a simple ratio of male death (or female death) by mortality cause over total deaths by that cause. Thus, the sum of all ratios should equal 1. From the ratios of mortality by gender in film and NVSS data, we constructed a bar graph visualization. Then, we explored the significance of the difference in gender ratio by cause of death between film and real life using the Pearson chi-square test [28,29].

It is important to underscore how to interpret significant *P* values in this context [30-32]. Accordingly, *P* values <.5 do not “measure the probability that the studied hypothesis is true, or the probability that the data were produced by random chance alone”, and are merely “one approach to summarizing the incompatibility between a particular set of data and a proposed model for the data” [30]. That is, if there is a significant *P* value for the chi-square test for any given cause of mortality, this means that the data seem to suggest there is a significant difference between expected and observed frequencies of causes of death by gender between film and real-life but does not establish causality.

### Ethical Considerations

All US mortality data used for this study are publicly available, anonymous, and consolidated at the national level. All data have been deidentified. Cinemorgue data comprise fictional, in-film deaths. This study is exempt from ethical approval, as the research involves the analysis of large-scale, publicly available datasets that are legally accessible to the public and do not require any special access or permissions. The data do not include any information through which individuals can be readily identified, either directly or indirectly. Because the data are both publicly available and do not involve interaction or intervention with human participants, nor do they involve the collection of private, identifiable information, this study qualifies for exemption from institutional review board oversight.

## Results

**Figure 1.**
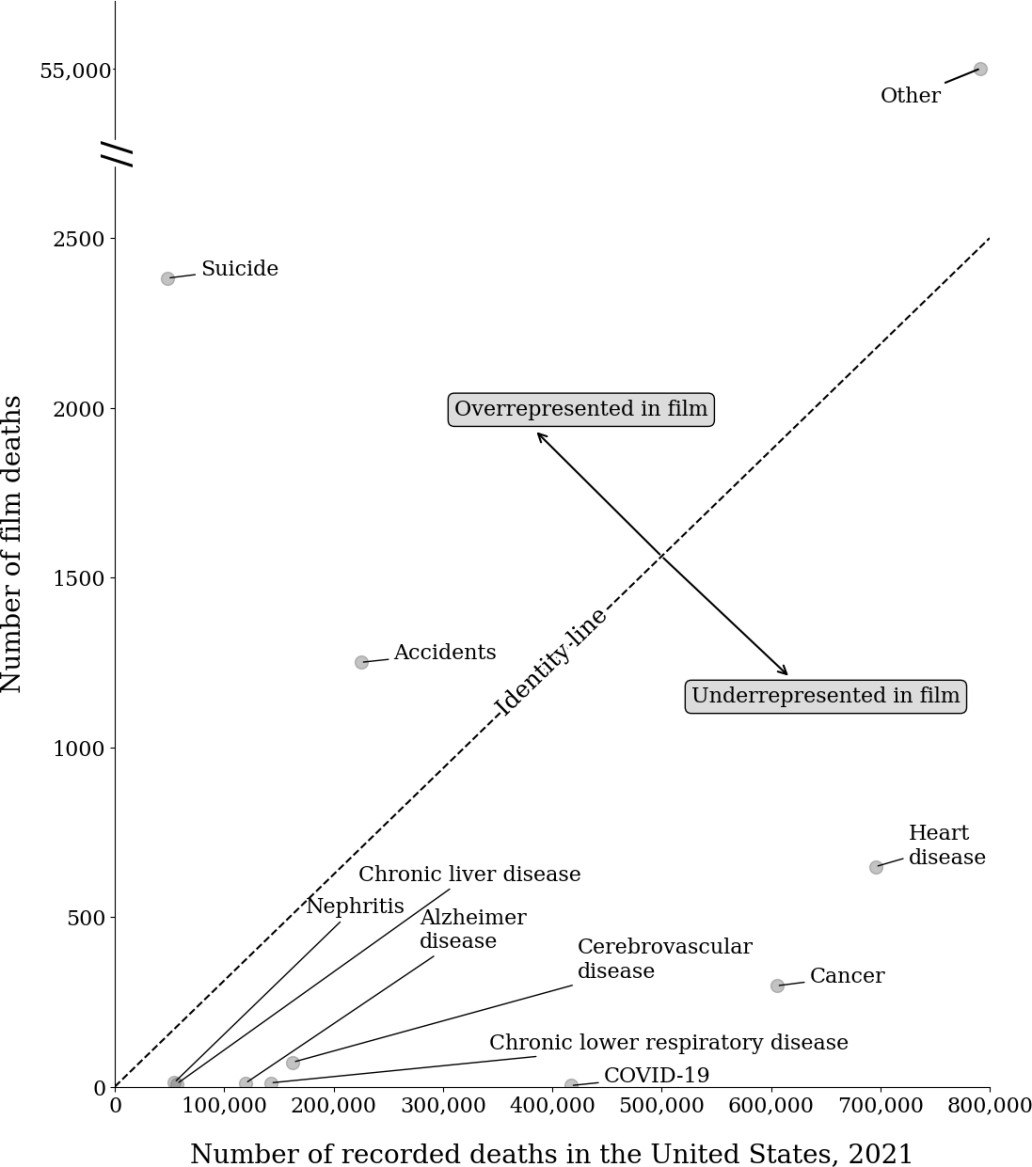
Overall number of total deaths in Cinemorgue versus number of recorded deaths in the United States, National Vital Statistics Survey 2021 by cause of mortality. Of total film deaths, suicide and accidents are overrepresented; all other causes of death are underrepresented in film. The identity line depicted the boundary between over- and underrepresentation; causes of death above the line are overrepresented, and causes of death beneath the line are underrepresented in film.

In general, the Cinemorgue dataset underrepresents most real-life leading mortality causes as compared with cinematic mortality causes by proportion of deaths in the database. There are 2 exceptions where suicide and accidents are overrepresented. Figure 1 depicts the identity line or line of no difference at which representation would be equal between both sources.

**Figure 2.**
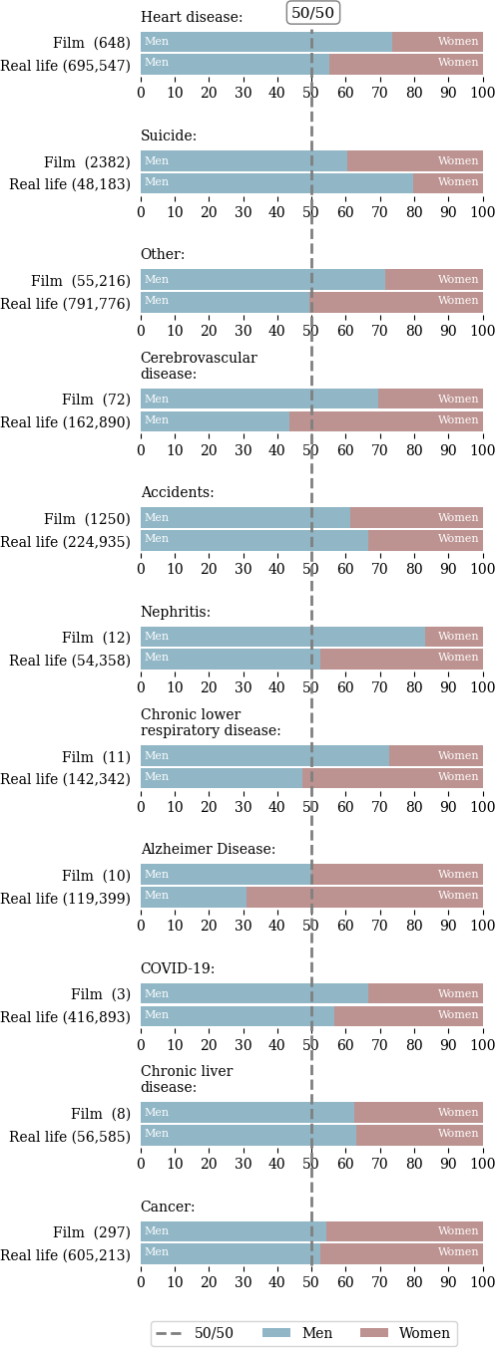
Top 10 mortality causes by gender in film versus real life. Count and proportion of deaths by gender (blue for men, mauve for women) are shown by mortality cause for film and for real life. Women suicide deaths and accidents are overrepresented in film. Men are overrepresented in all other causes of death. We only included causes of death present in the Cinemorgue dataset.

When considering gender in addition to raw count by database, the differences between film and real life are also apparent. Figure 2 is a subplot panel depicting the relative proportions of 2021 NVSS leading causes of mortality by gender versus all-time film deaths by gender from Cinemorgue. Across almost all causes of death except cancer and chronic liver disease, there are visible and apparent shifts between film representation and real-life representation, with women underrepresented in film across nearly all categories except for suicide. Diabetes and essential hypertension are not represented in the cleaned Cinemorgue dataset. The counts used to construct the ratio shift figure are contained in Table S2 in Multimedia Appendix 1.

In Table 4, we show the significance (if observed frequency *>*5) of the ratio shift for each cause of death. The ratios by gender of accidents, cerebrovascular disease, diseases of the heart, suicide (intentional self-harm), and the combined category of all other causes of mortality for film deviates significantly from that in real life. As previously mentioned, men are overrepresented in film as compared with women for each of the aforementioned causes of mortality, barring suicide.

**Table 4.** Chi-square test: statistic and *P* values. Each cause of death and its corresponding chi-square test by gender proportion is depicted, with its significance value.

Cause of death	Chi-square (*df*)	*P* value
Accidents^a^	15.81 (1)	<.001
Alzheimer disease	—^b^	—
COVID-19	—	—
Cancer	0.29 (1)	.59
Cerebrovascular disease^a^	19.72 (1)	<.001
Chronic liver disease	—	—
Chronic lower respiratory diseases	—	—
Diseases of heart^a^	87.57 (1)	<.001
Nephritis	—	—
Other^a^	10,832.92 (1)	*<*.001
Suicide^a^	545.54 (1)	*<*.001

a Significant findings (where film deaths by gender are not representative of real life). There is a significant difference in representation for accidents, cerebrovascular disease, diseases of the heart, suicide, and all other causes.

b Cause of death categories in which there were less than 5 observations in films, and thus the chi-square test was not appropriate [25].

## Discussion

### Principal Findings

Film and real-life mortality differ significantly for some, but not all, leading causes of death. As expected, film is not representative of real life even setting aside gender considerations. From a practical perspective, this makes sense in the context that high-tempo, salacious, or dramatic deaths are likely far more interesting to an audience than everyday causes of mortality. In film, for example, the proportion of “other” deaths represented far outpaces the actual leading cause of death (diseases of the heart) at a factor of over 800. When considering gender, there are significant differences in the ratio of represented death for accidents, cerebrovascular disease, diseases of the heart, suicide (intentional self-harm) and all other deaths not captured using the top 10 leading causes of death classifier, among sufficiently large samples within the Cinemorgue Wiki database.

While our results do not indicate causality, they provide insight to the representation of mortality in popular media. From visualizations and statistical comparison of ratios, there is meaningful descriptive power in the ratios of film versus real-life representation of mortality by gender. In view of recent efforts by the American Heart Association to provide better equality of cardiovascular care for women [3] as well as general research regarding treatment differences by gender [33], the underrepresentation of film deaths by gender demonstrate that death is also lacking in equitable representation on screen. As there is a general consensus in the literature that stereotypes can be exceedingly harmful and contribute to sexism, harassment, and violence against women [13] as well as other gender identities [34], it is reasonable to postulate that a lack of representation in film could exacerbate the public’s misunderstanding of disease burden and processes, and could hinder the recognition of gender-specific health issues among not only the public but potentially among health care professionals.

By cause of mortality, some interesting patterns emerge. For the category of “other,” men are overrepresented in the Cinemorgue Wiki, which is consistent with their overrepresentation writ large in the Wiki (regardless of cause of death). This could indicate that men are represented more often on screen generally, and represented more often in violent deaths, as previously discussed. For diseases of the heart, men are seemingly overrepresented in film, which agrees with the American Heart Association’s recent findings on underrepresentation and lack of understanding of women experiencing heart disease [3]. Likewise, men are overrepresented for death by cerebrovascular disease. However, suicide and accidents are overrepresented for women in film. Based on the suggestibility and risk associated with suicide representations [14], this pattern indicates that future follow-up is needed to understand the relationship between representation and acts of suicide in women.

### Limitations

Limitations of this study center on the classification and cleaning of the Cinemorgue film death dataset, as well as the data itself. First, we used only film deaths in this study, on the assumption that films generally have wider ranges of audiences (not confined to certain streaming platforms) and have a similar format. However, the Cinemorgue Wiki database includes all manners of video-represented deaths, including television and video games, which were excluded in this study based on the assumption that video game deaths may overrepresent homicides, due to the violent nature of many video games. Furthermore, we compare the Cinemorgue Wiki database all-time counts with single point-in-time (2021 NVSS) mortality counts; considering that movies are not only watched in the year in which they are filmed, we consider this a reasonable assumption even though movie popularity changes over time. In addition, in Table 1, we report that 50% (34,268/68,535) of the film deaths in our dataset are from 1995 and later, with 25% (17,132/68,535) occurring between 2010 and 2023, showing that the preponderance of films occur relatively close to the point-in-time mortality counts with many being topically contemporary to the year in which they were filmed.

In addition, our removal of androgynous names may have skewed the resultant data; as illustrated previously in Table 2, 13% of data are effectively “thrown out” of the analysis because it was not classifiable as “female” or “mostly female” or “male” or “mostly male”. While using binary categories is consistent with the CDC’s current reporting causes by mortality, reducing gender to solely men and women does not capture the diversity of gender identities. Considering the connection between media representation and suicide, as well as the significantly higher instances of suicidal ideation in transgender and nonbinary youth than cisgender youth [35], indicates that further research beyond this study is important for understanding the representation of health outcomes for transgender and nonbinary individuals. In terms of classifying cause of mortality in the Cinemorgue Wiki database, the search terms used on the stemmed text were specific but very broad in order to prevent an undercount of deaths, which could have skewed resultant counts. Intrinsically, the Cinemorgue Wiki database is also subject to the normal risks of crowd-sourced data. Members of the public that are heterogeneous in knowledge, skill, and geographical location comprise the crowd [36] that generates the entries in the Cinemorgue Wiki; biases in any of these characteristics could contribute to a geographically skewed or low-fidelity dataset. While CDC data from the United States were used to calculate gender proportions, every film death in the database was included, even if the film was not English-language. Accordingly, while some films may better represent certain realities than others, research has shown that even explicitly far-fetched or out-of-reality story lines can influence beliefs via transportation [37], so we have retained all films regardless of location of origin or setting.

### Future Work

Future research should further detail the corpus of cinematic mortality, and incorporate additional features of each film (eg, age of actors, setting, location, audience scores, and critic reviews) from film encyclopedias and rating websites. This will further elucidate the nature of mortality representation in film relative to viewership. In addition, future work should examine mortality representation across all forms of media, including books, songs, and social media. This would provide a more global perspective on the impact of media—writ large—and capture populations that may not interact with cinematic media. With enough data, the Granger causality test—used to forecast time series from other time series [38]—could be used to understand whether media representation of death by gender lags or leads actual causes of mortality by gender. This would contribute to a better understanding of the interplay between media representation and health outcomes over time. Relatedly, visualizations like a Sankey flow diagram—which provides visualization of time-series-type data in lieu of a formal time series analysis [39]—could provide further opportunities for interpretation of how gender representation has morphed over time. Randomized controlled trials centering on these ideas (perhaps priming viewers with biased images of disease before surveying them on perceived prevalence of disease by gender) could provide support for the case of causality between representation and understanding of society’s disease burden.

### Conclusions

Our study compared mortality causes by gender in film and real life using Cinemorgue Wiki data on all-time film deaths and NVSS mortality by cause data from 2021 in the United States. Based on the comparison of gender ratios by cause of death, we identified significant differences in representation for accidents, cerebrovascular disease, diseases of the heart, and suicide (intentional self-harm). Considering current struggles in appropriately managing diagnosis and treatment of disease by gender, highlighting how representation of mortality is skewed by gender in films may encourage a more equitable culture surrounding gender and health. Future work should expand this query to include other forms of media to understand the interplay of representation and mortality.

## Supplementary material

10.2196/70853 Multimedia Appendix 1 All film counts and percentages for causes of mortality in the Cinemorgue database.

## Abbreviations

CDC: Centers for Disease Control and Prevention
*ICD-10*: *International Classification of Diseases, Tenth Revision*
NVSS: National Survey of Vital Statistics
