# Supplementary material for: Misrepresentation of Overall and By-Gender Mortality Causes in Film Using Online, Crowd-Sourced Data: Quantitative Analysis
Source: JMIR Form Res. 2025 Jun 24;9:e70853. doi: 10.2196/70853 (PMC12212890; doi:10.2196/70853)
Supplement: Multimedia Appendix 1 [file formative-v9-e70853-s001.docx]

**Table S1.** Percentage and Count for Leading Causes of Mortality in the United States in Cinemorgue Wiki Database.

| **Mortality Causes** | **Total** | **Female %** | **Female**  **Count** | **Male %** | **Male**  **Count** |
| --- | --- | --- | --- | --- | --- |
| 1. Diseases of the Heart | 648 | 26.4% | 171 | 73.6% | 477 |
| 2. Malignant Neoplasm *(Cancer)* | 297 | 45.8% | 136 | 54.2% | 161 |
| 3. COVID-19 | 3 | 33.3% | 1 | 66.7% | 2 |
| 4. Accidents (unintentional injuries) | 1,250 | 38.8% | 485 | 61.2% | 765 |
| 5. Cerebrovascular Disease *(stroke, brain hemorrhage)* | 72 | 30.6% | 22 | 69.4% | 50 |
| 6. Chronic Lower Respiratory Disease *(COPD, emphysema, etc.)* | 11 | 27.3% | 3 | 72.7% | 8 |
| 7. Alzheimer Disease | 10 | 50% | 5 | 50% | 5 |
| 8. Diabetes Mellitus | — | — | — | — | — |
| 9. Chronic Liver Disease | 8 | 37.5% | 3 | 62.5% | 5 |
| 10. ‡Nephritis | 12 | 16.7% | 2 | 83.3% | 10 |
| †Suicide (intentional self-harm) | 2,382 | 39.7% | 945 | 60.3% | 1437 |
| ‡Essential hypertension | — | — | — | — | — |
| ˆOther | 55,216 | 28.4% | 15,678 | 71.6% | 39,538 |

†Denotes *top 10* cause of death for males only

‡Denotes *top 10* cause of death for females only

ˆ“Other” causes of death are all deaths not captured in the categories above

**Table S2.** Percentage of Leading Causes of Mortality in the United States in Cinemorgue Wiki Database versus NVSS.

| **Mortality Causes** | **Cinemorgue** | **NVSS** |
| --- | --- | --- |
| 1. Diseases of the Heart | 1% | 19.6% |
| 2. Malignant Neoplasm *(Cancer)* | *<*1% | 17.1% |
| 3. COVID—19 | *<*1% | 11.8% |
| 4. Accidents (unintentional injuries) | 2.1% | 6.4% |
| 5. Cerebrovascular Disease *(stroke, brain hemorrhage)* | *<*1% | 4.6% |
| 6. Chronic Lower Respiratory Disease *(COPD, emphysema, etc.)* | *<*1% | 4.0% |
| 7. Alzheimer Disease | *<*1% | 3.4% |
| 8. Diabetes Mellitus | — | 2.9% |
| 9. Chronic Liver Disease | *<*1% | 1.6% |
| 10. ‡Nephritis | — | 1.5% |
| †Suicide (intentional self—harm) | 3.9% | 1.4% |
| ‡Essential hypertension | — | 1.21% |
| ˆOther | 92.4% | 24.9% |

†Denotes *top 10* cause of death for males only

‡Denotes *top 10* cause of death for females only

ˆ“Other” causes of death are all deaths not captured in the categories above
